# Interdisciplinary Health Care Evaluation Instruments: A Review of Psychometric Evidence

**DOI:** 10.1177/01632787211040859

**Published:** 2021-08-19

**Authors:** Hosung (Joel) Kang, Cecilia Flores-Sandoval, Benson Law, Shannon Sibbald

**Affiliations:** 1 Faculty of Health Sciences, University of Western Ontario, London, Ontario, Canada; 2 Health and Rehabilitation Sciences, University of Western Ontario, London, Ontario, Canada; 3 Department of Family Medicine, Schulich School of Dentistry and Medicine, University of Western Ontario, London, Ontario, Canada

**Keywords:** review, interdisciplinary collaboration, teamwork, surveys, evaluation

## Abstract

Teamwork among health care professionals has been found to improve patient outcomes and reduce burnout. Surveys from individual team members are often used to measure the effectiveness of teamwork performance, as they provide an efficient way to capture various constructs of teamwork. This allows evaluators to better understand team functioning, areas of strength, and to identify potential areas for improvement. However, the majority of published surveys are yet to be validated. We conducted a review of psychometric evidence to identify instruments frequently used in practice and identified in the literature. The databases searched included MEDLINE, EMBASE, CINAHL, and PsycINFO. After excluding duplicates and irrelevant articles, 15 articles met the inclusion criteria for full assessment. Seven surveys were validated and most frequently identified in the literature. This review aims to facilitate the selection of instruments that are most appropriate for research and clinical practice. More research is required to develop surveys that better reflect the current reality of teamwork in our evolving health system, including a greater consideration for patient as team members. Additionally, more research is needed to encompass an increasing development of team assessment tools.

Health care professionals are increasingly being asked to work in more complex ways with less resources (Palumbo, 2017; Rosser et al., 2011). One approach to meet this demand is for health care professionals to work in collaborative interprofessional teams. Interprofessional teams have been shown to improve patient outcomes, decrease service duplication, and reduce health care providers’ feelings of burnout (O’Leary et al., 2010). Team-based care approaches are common across all levels and sectors of health care. With primary care in Ontario, the Ministry of Health introduced interprofessional groups called Family Health Teams (FHTs) to bring together physicians and allied health care professionals (Hutchison & Glazier, 2013; Rosser et al., 2011). Collaboration is strongly encouraged within hospital settings including, but not limited to, emergency departments, operating rooms, and neonatal resuscitation areas. This approach to health care has been found to reduce errors, improve quality of care and patient outcomes, reduce health care workloads and cost, and increase job satisfaction and retention (Boult et al., 2001; Buist et al., 2002; Langhorne & Duncan, 2001; Morey et al., 2002).

Despite the increasing demand on teams to deliver care, there is a lack of consensus on how to measure the effectiveness of a health care team. The most commonly used method is through surveys from team members (Valentine et al., 2015), which allow for an efficient method of collecting data that can be easily interpreted (Brinkman et al., 2006). Surveys usually assess several dimensions of teamwork such as communication, cohesion, and role clarity, by providing a score on each dimension. As an integral part of interprofessional collaboration interventions in clinical settings, surveys facilitate the measurement of pre- and post-evaluation variables. These scores can then be analyzed (using a statistical software) to detect changes as a result of the intervention (Gellis et al., 2019).

Over the past few decades, there have been a number of instruments with varying degrees of psychometric properties developed to measure teamwork in health care settings (Valentine et al., 2015), and a wide range of validated and unvalidated tools (Strating & Nieboer, 2009). Systematic reviews have been conducted to summarize the available literature and tools. Some reviews focus on specific health care settings, to identify validated instruments that could be used to measure teamwork within a specific context (Walters et al., 2016). Researchers often still choose to create their own surveys *de novo*, despite consistent recommendations found in the peer review literature to modify existing surveys instead of creating a new instrument. Given the vast number of instruments available, coupled with the increasing pressure to demonstrate value in health care, it is now more important than ever to identify surveys with a record of construct and content validity that can be applicable to specific health care settings. We conducted a review to: (1) identify instruments and their psychometric evidence, and (2) provide an overview of the properties, limitations, and theoretical underpinnings of these instruments. Our aim was to support health care professionals and researchers seeking to choose the most appropriate instrument to evaluate teamwork within the context of their practice.

## Method

### Search Strategy

A systematic literature search was performed in consultation with a health sciences research librarian to identify relevant reviews of instruments to measure teamwork within a health care setting. Our literature search strategy used key words that described teamwork such as “team,” “interprofessional collaboration,” “interprofessional relations [MESH Terms]” with “surveys,” “questionnaires,” “measurement” and “assess.” The selected databases were MEDLINE, EMBASE, CINAHL, and PsycINFO. The search strategy was adapted to meet the specific requirements of each database and was limited to only review articles and available in English-language, published from January 2000 to September 2017. The following search was used: Search (evaluation OR evaluate OR assessment OR assess OR measurement OR measure OR instrument OR instruments OR questionnaires OR surveys) AND ((health care team[MeSH Terms]) OR “multiprofessional collaboration” OR “interdisciplinary collaboration” OR (interprofessional relations[MeSH Terms]) OR “team-based” OR “interprofessional collaboration” OR team OR teamwork) AND review[Title]. When possible, articles published in “review” type format were searched as opposed to the entire directory to increase fidelity and to limit identification of irrelevant papers. Forward and backward searches were done with a leading review article by Valentine and colleagues (2015), a seminal review article that identified instruments related to health care teams.

In line with our inclusion criteria, articles must have contained a review of surveys or instruments used in assessing teamwork in any health care setting to be included. Given that they present the highest level of evidence, we only considered systematic reviews. Articles were deemed to be systematic reviews not only if they self-identified as one, but also if they met the stringent methodological requirements set forth by PRISMA or another validated checklist tool. We excluded review articles that summarized theories or concepts of teamwork, or articles that were published within the interprofessional education context. Results from the database and forward/backward searchers were reviewed by three independent reviewers (HK, CFS, RV) who read the title and abstracts to narrow down the selection. The final selection of articles was achieved through multiple meetings and discussion with the research team.

### Quality Assessment

Concerning quality assessment, we used the Risk of Bias in Systematic Reviews (ROBIS) checklist (Whiting et al., 2016). The ROBIS is a checklist for the assessment of the risk of bias in systematic reviews. ROBIS has three distinct phases in assessing a review. As the systematic reviews in our study do not include participants or interventions, phase one, which is used to assess relevance by identifying participants, interventions, comparisons, and outcomes (PICO), which is optional, was deemed unnecessary for the purpose of this study. Phase 2 (identify areas where bias may be introduced into the systematic review) and 3 (consider whether the systematic review as a whole is at risk of bias) were completed to assess risk of bias. Phase 2 involved the assessment of four domains to cover key review processes: study eligibility criteria; identification and selection of studies; data collection and study appraisal; and synthesis and findings. Phase 3 assessment uses the same structure as the phase 2 domains, including signaling questions and information used to support the judgment, but the judgment regarding concerns about bias is replaced with an overall judgment of risk of bias. Two independent reviewers (HK and CFS) used the checklist for each article. Any discrepancy was discussed within the research team to reach a consensus.

In addition, the ROBIS checklist was the most suitable checklist for quality assessment as it accounts for reviews misappropriating themselves as a systematic review. Specifically, domain 3.4 of the ROBIS checklist addresses methodological concerns of the aforementioned systematic reviews.

### Search Results

After all relevant systematic reviews were identified, detailed information was extracted into a Microsoft Excel spreadsheet with the following categories: purpose of the review, applicable health care setting, dimensions of teamwork, search strategy, theoretical framework that guided the search, risk of bias assessment, list of instruments (validated and unvalidated). In a different Excel sheet, the instruments identified from the reviews were aggregated into a master list, which included the frequency count for each instrument. The surveys that appeared more frequently in the selected literature were identified by counting the number of times in which the survey was mentioned in the reviews. Counting the frequency yielded a good, but imperfect, measure of robustness (Aksnes et al., 2019). Instruments that were identified four times across reviews were deemed “robust” for the purpose of this study (see Table 1: Psychometric Properties). We only included instruments that appeared in the master list at least four times. Instruments’ psychometric properties, dimensions of teamwork, theoretical underpinnings, number of questions, and its applicability in various health care settings were reported. Psychometric properties, such as internal consistency, inter-rater agreement and reliability, and validity, were reported for the selected instruments when the information was available.

**Table 1.** Psychometric Properties.

Author	Name of Instrument	Number of Questions	Likert Scale (5 or 7 Point)	Attributes of Teamwork	Reliability	Internal Consistency	Validity	Theoretical Base
Schroder et al. (2011)	Collaborative Practice Assessment Tool (CPAT)	56 3 Qualitative Questions	7	*Mission *Meaningful purpose*Goals *General relationships *Team leadership *General role *Responsibilities and autonomy *Communication and information exchange *Decision-making and conflict management *Community linkages and coordination of care *Patient involvement		Pilot test #1—EFA seven domains; 42 items Cronbach’s α = .73–.84 Pilot test #2 CFA—56 items; eight domains Cronbach’s α =.67–.89 Overall score (α =.95) Cronbach’s α = .72–.92 for domains	Face and content validity EFA and CFA in pilot tests with positive results	Based on constructs of collaboration identified in the literature and a review of existing tools to assess perceptions of teamwork and collaboration in health care
Oliver et al. (2007)	Modified Index of Interdisciplinary Collaboration (MIIC)	42	5	*Interdependence *Flexibility *Newly created professional activities *Collective ownership of goals *Reflection on process	Original IIC—Test– retest correlation was .824 (p < .01)	Original IIC, overall Cronbach’s α = .92 and all subscales Cronbach’s α over .75 MIIC—overall Cronbach’s α = .935 Subscales range .77–.87 (Kobayashi & McAllister, 2013: Parker Oliver et al., 2007)	CFA with four subscales	Based on Bronstein’s model of interdisciplinary collaboration (2003) based on four theoretical perspectives
Cooper et al. (2010)	Team Emergency Assessment Measure (TEAM)	11 items	5	*Leadership *Global perspective *Communication *Working together in tasks *Composure and control	Intraclass correlation coefficient of the global score was 0.93	Internal consistency (Cronbach’s α) of 0.89	Content validity is high, with a content validity index of 0.96	
Shortell et al. (1991)	ICU Nurse Physician Collaboration	82	5 point	*Communication *Use of expertise *Coordination *Shared decision-making *Active conflict management *Effort *Respect	Reliabilities from 0.66 to 0.92	α = 0.62–0.9	7 Factor Model confirmed by CFA	
Anderson and West (1998)	Team climate inventory	38	7/5 points	*Shared workload *Shared decision-making *Communication *Coordination *Collaboration *Use of expertise *Respect *Group cohesion *Shared objectives *Social support *Psychological safety	The reliability of the total scale was 0.76.	Cronbach’s αs 0.88 to 0.93	Exploratory factor analysis confirmed the original four-factor model. Higher performance on the TCI has been associated with improved health outcomes better access to care, improved patient satisfaction and improved job satisfaction and openness to innovation.	Based on four-factor theory of climate for innovation
Undre et al. (2007)	OTAS (Observational Teamwork Assessment for Surgery)	45	7	*Communication *Communication *Coordination *Cooperation/backup behavior *Leadership *Monitoring/awareness	Observer agreement was high (Cohen’s κ ≥ 0.41)		Validity achieved by expert practitioners’ consensus and expert panels	
Malec et al. (2007)	MHPTS (Mayo High Performance Teamwork Scale)	16	3	*Recognizing the leader *Balance between authority and team member participation *Clear understanding of roles *Involvement with the patient *Conflict solution and situation awareness		Cronbach’s α = 0.85	Construct validity by Rasch (person reliability = 0.77	

*Note*. Empty cell represents unknown information.

**Figure 1.**
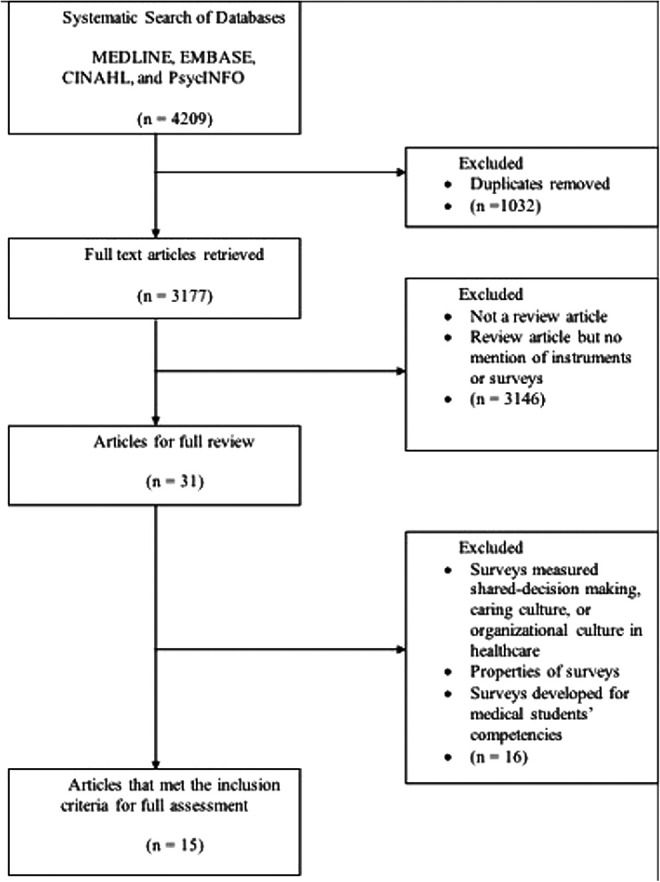
Literature search results.

The search generated 4,209 potentially relevant articles from multiple disciplines including nursing, medicine, and social sciences (See Figure 1). After duplicates were removed, 3,177 articles remained. Three independent reviewers read through the title and abstract. The vast majority of the articles were excluded because they were not a review article or because they described theories of teamwork without mentioning surveys or instruments. There were 31 potential articles remaining. From the 31 articles, 16 were excluded because the dimensions that guided the review were not relevant to teamwork in an interdisciplinary health care setting, failed to expand on details other than the conceptual framework of instruments, or because instruments were mentioned in interprofessional education context. The selected 15 review articles reported a list of instruments to a specific context or a health care setting within their own purpose of research.

## Results

### Health Care Setting

The objectives of the included articles varied widely. Some articles aimed to identify instruments for a specific health care setting whereas other studies provided a comprehensive review of instruments intended to measure interprofessional collaboration in general, without focusing on a particular health care setting. Bookey-Basset and colleagues (2016) aimed to identify instruments that measure interprofessional collaboration in the context of chronic disease management among community dwelling older adults and to determine the strengths and limitations of such instruments. Three review articles aimed to identify instruments that assess team effectiveness in obstetric emergencies (Clary-Muronda & Pope, 2016; Fransen et al., 2017; Onwochei et al., 2017). Among these three articles, one article primarily looked at instruments appropriate to the measurement of teamwork in neonatal resuscitation teams (Fransen et al., 2017). One article aimed to identify instruments measuring teamwork in surgery (Whittaker et al., 2015), another aimed to identify instruments measuring teamwork in internal medicine (Havyer et al., 2014), and one in medical education (Havyer et al., 2016). Two articles by Cooper, Cant, et al. (2010) and Cooper, Endacott, and Cant (2010) provided a review of instruments that measured non-technical skills to assess teamwork in medical emergencies. One review article aimed to identify teamwork in health care action teams (Rosenman et al., 2015). There were six articles that looked at instruments that measure interprofessional collaboration, not focusing on any specific health care setting (Dougherty & Larson, 2005; Jacob et al., 2017; Schroder et al., 2011; Shoemaker et al., 2016; Valentine et al., 2015; Walters et al., 2016).

### Teamwork Dimensions

Not surprisingly, dimensions of teamwork overlapped across many different reviews. The teamwork dimensions that were frequently mentioned were: communication, cooperation, coordination, leadership, and situational awareness. Less frequently identified dimensions included use of expertise, conflict management, newly created professional activities, social support, psychological safety, and organization culture. Out of 15 articles, only two articles included patient involvement as a teamwork dimension (Havyer et al., 2016; Schroder et al., 2011). The dimensions identified in each article were primarily determined by the type of theoretical underpinnings of collaborative practices. For instance, tools like the Partnership Self-Assessment tool are based on the partnership synergy framework, putting emphasis on the key indicators of successful collaboration (Lasker et al., 2001), whereas tools such as the Edmondson tool gives attention to psychological safety as the main dimension in understanding teamwork performance (Edmondson, 1999).

### Methodological Quality Assessment Use

Most of the systematic reviews that were assessed used the standard PRISMA guidelines to synthesize the data (Moher et al., 2009). Others have also included using the COSMIN checklist (Consensus-based standards for the selection of health measurement instruments; Mokkink et al., 2010). Both COSMIN Study Design checklist and COMINS Risk of Bias checklist were used by the selected reviews to assess methodological quality of their included studies.

Shoemaker and colleagues (2016) used the input-process-output framework of team-based primary care (Rydenfält et al., 2017) to guide the identification and assessment of available measurement instruments. The conceptual framework presents inputs, mediators, and outputs of effective teamwork in primary care. “Inputs” refer to precursors or pre-conditions that make it possible for teams to exist. “Mediators” are processes that occur within the team. “Outputs” are the results of effective teamwork. Mediators include cognitive (sense-making, continuous learning, shared explicit goals and accountability, and evolving mental models of roles), affective/relational (trust, respectful interactions, heedful inter-relating, and commitment), behavioral (communication, adaptable to context and needs, and conflict resolution), and leadership domains that contribute to effective teamwork.

The Oxford’s Center for Evidence Based Medicine (OCEBM) guideline was also used. This tool aims to facilitate the process of finding appropriate evidence, to help make results explicit and to assess the evidence (Durieux et al., 2013). The Social Ecological Model (SEM) was also used to guide one integrative review (Clary-Muronda & Pope, 2016).

### Risk of Bias

The systematic reviews included in this paper had very low risk of bias as assessed by the ROBIS checklist. Each review had clearly defined inclusion/exclusion criteria, the searches were appropriate given that a wide range of databases were searched, and authors clearly defined what guidelines or models they used to guide the research. Some authors employed forward and backward searches of seminal articles to further search the literature and thereby increase credibility. To reduce the risk of bias, most reviews adhered to PRISMA guidelines or other quality assessment guidelines. Additionally, risk of bias was reduced by having multiple reviewers assess the inclusion and exclusion criteria of articles. In the case of Cooper and colleagues (2010; 2013) the synthesis and findings were deemed unclear because the low number of articles identified in its initial data search made it difficult to assess whether the authors found all relevant articles.

### Interprofessional Teamwork Instruments

Sixteen instruments were frequently identified, seven of which received the most attention in the literature: Collaborative Practice Assessment instrument (CPAT), Mayo High Performance Teamwork Scale (MHPTS), Modified Index for Interdisciplinary Collaboration (MIIC), Intensive Care Unit Nurse-Physician Questionnaires (ICU N-P-Q), Observational Teamwork Assessment for Surgery (OTAS), Team Climate Inventory (TCI), and Team Emergency Assessment Measure (TEAM). See Table 1 for psychometric properties and Table 2 for an overview of the seven instruments. We provide a comprehensive description of each instrument below. As well, the face validity, a key metric of psychometric validity (Litwin & Fink, 2003), is examined.

**Table 2.** Overview of Tools.

Tool	Overview
Collaborative Practice Assessment instrument (CPAT)	– General tool and applicable to a variety of clinical settings. – 56 closed ended question on a 7-point Likert scale. – Domains: mission, meaningful purpose, goals, general relationships, team leadership, general role responsibilities and autonomy, communication and information exchange, decision-making and conflict management, community linkages and coordination of care, and patient involvement.
Mayo High Performance Teamwork Scale (MHPTS)	– Contains 16 questions. – Explores explicit goals and accountability, heedful interrelating, communication, adaptability, conflict resolution, and leadership.
Modified Index for Interdisciplinary Collaboration (MIIC)	– Founded on four perspectives: a multidisciplinary theory of collaboration, services integration, role theory, and ecologic systems theory. – Six components of collaboration: interdependence, newly created professional activities, flexibility, collective ownership of goals, and reflection on process.
Nurse Physician Collaboration (ICU)	– Catered towards working relationship between nurses and physicians. – Original version had 120 items on a 5-point Likert scale. – Revised version available with 81 items. – Measures organizational climate, with a focus on unit culture, leadership, communication, coordination, problem-solving and conflict management.
Observational Teamwork Assessment for Surgery (OTAS)	– Catered towards teamwork in a surgical environment. – Fifteen items on a 7-point Likert scale. – Five dimensions: communication, coordination, cooperation and back up behavior, leadership, team monitoring and situational awareness.
Team Climate Inventory (TCI)	– Grounded in the four-factor theory of climate for innovate: participative safety, support for innovation, vision and task orientation. – 38 item self-report questionnaire.
Team Emergency Assessment Measure (TEAM)	– Covers three core categories (leadership, teamwork and task management) and nine elements (leadership control, communication, team climate, adaptability, situation awareness, prioritization, clinical standards, co-operation and co-ordination). – 11 questions on a 5-point Likert scale and one question using a global rating, totaling 12 questions.

#### Collaborative Practice Assessment Tool (CPAT)

The CPAT was first developed at Queen’s University and funded by Health Canada (Paterson et al., 2013). CPAT is composed of 56 closed ended questions based on a 7-point Likert scale with three additional open-ended questions to gain further insight of teamwork performance. The teamwork domains included in the instrument are: mission, meaningful purpose, goals, general relationships, team leadership, general role responsibilities and autonomy, communication and information exchange, decision-making and conflict management, community linkages and coordination of care, and patient involvement.

The CPAT provides good insight as to which dimensions of teamwork need improvement and where the team is lacking. The CPAT was developed to assist health care professionals in identifying strengths and weaknesses in their collaborative practice, thereby providing opportunities for improvement in their clinical practice (Schroder et al., 2011). The design of the instrument was based on dimensions of collaboration identified in the literature and a review of existing instruments to assess perceptions of teamwork and collaboration in health care. The instrument was intended to be general in nature in order to allow for flexibility and application across a wide variety of clinical practice settings and with a range of health care providers (Schroder et al., 2011). The overall result from the two pilot tests indicated that the CPAT is a valid and reliable tool for measuring health care team members’ perceptions of working collaboratively (Schroder et al., 2011). In assessing levels of collaborative practice within teams, it provides a basis upon which teams can begin to explore domains that would benefit from educational interventions.

#### Mayo High Performance Teamwork Scale (MHPTS)

The MHPTS was designed to be short and to be used by participants in training and by team members in other settings to rate key behaviors of high-performance teams (Malec et al., 2007). This instrument can be used to assess a team’s high-performance teamwork and crisis resource management skills in a simulation setting. There are 16 questions that ask about shared explicit goals and accountability, heedful interrelating, communication, adaptability, conflict resolution, and leadership. There is evidence of satisfactory reliability and initial support for the construct validity, however further evaluation is required to assess its validity in various educational and clinical settings. Nevertheless, the instrument shows signs of promise as it has recently been translated to different languages and shows acceptable psychometric properties when rigorously tested on nursing students (Gosselin et al., 2019).

#### Modified Index for Interdisciplinary Collaboration (MIIC)

Bronstein originally developed the Index for Interdisciplinary Collaboration instrument to measure social workers’ perception of interdisciplinary collaboration (Oliver et al., 2007). The MIIC was later created to include other health care professionals in the design of the instrument. The conceptual framework for this instrument was developed based on four theoretic perspectives: a multidisciplinary theory of collaboration, services integration, role theory, and ecologic systems theory. The model identifies five components of collaboration: interdependence, newly created professional activities, flexibility, collective ownership of goals, and reflection on process. MIIC has demonstrated a capacity to measure and differentiate variances in the perception of collaboration within a hospice setting and to measure collaboration in expanded school mental health programs (Oliver et al., 2007).

#### Intensive Care Unit Nurse-Physician Questionnaires (ICU N-P-Q)

The ICU Nurse-Physician questionnaire was first developed by Shortell and colleagues (1991) and has been modified throughout the years by different researchers. The assumption of the questionnaire is that the nurses and physicians work in relational coordination (i.e., high-quality relationships exemplified by shared goals, shared knowledge, and mutual respect). The instrument measures organizational climate, with a focus on unit culture, leadership, communication, coordination, problem-solving, and conflict management. The original ICU N-P-Q is a 120-item scale derived from the Organizational Culture Inventory with response items ranked on a 5-point Likert scale ranging from 1 = strongly disagree to 5 = strongly agree. A revised and shortened version of the instrument is also available as an 81-item scale. The scale includes separate questionnaires for physicians and nurses. Shortell and collaborators (1991) reported that Cronbach’s α reliabilities ranged from 0.61 to 0.88 for subscales (acceptable in exploratory research). Other researchers have reported reliabilities from 0.66 to 0.92.

#### Observational Teamwork Assessment for Surgery (OTAS)

The OTAS instrument consists of five behaviors that team members in the operating room exhibit during surgery (Undre et al., 2007). Taken together, these behaviors provide an index of the quality of interprofessional teamwork in the operating room. The five behavioral dimensions of teamwork are communication, coordination, cooperation and back up behavior, leadership, team monitoring and situational awareness. This instrument can be used in real-time observation in the operating room or a relevant video recording of a surgery. The questionnaire has 15 items on a 7-point Likert scale (from 0 to 6), where 6 means exemplary behavior and very highly effective in enhancing team function whereas 0 means problematic behavior and team function is severely hindered.

The OTAS tool considers the variety of health care professionals that work in operating rooms, including surgeons, anesthetists, and nurses (scrub nurses and circulating nurses), who work together to provide the best patient care. Because of this, the observer provides separate behavioral scores for each of the three sub-teams: the surgical sub-team (and assistants), the anesthetic sub-team (anesthetist and anesthetic nurse), and the nursing sub-team (scrub nurse/practitioner and circulating nurses).

#### Team Climate Inventory (TCI)

The TCI instrument was developed by organizational psychologists to evaluate team functioning (Anderson & West, 1998). Team climate has been conceptualized as one of two (overlapping) approaches: the cognitive schema approach focuses on the relationship amongst environment, attitudes and behaviors, while the shared perceptions approach focuses more on aggregate perceptions of the environment. TCI is based on four-factor theory of climate for innovation: (a) *participative safety* acknowledges that trust is essential for members’ involvement; (b) *support for innovation* is the expectation of and support for the introduction of new ways of doing things; (c) *vision* refers to valued outcomes and a common higher goal as motivating factors; and (d) *task orientation* refers to a shared concern for excellence (Anderson & West, 1998). There are several different variations of the TCI tool with a different number of questions and versions that have been adapted to a variety of languages.

The four-factor model is based on vision, participative safety, task orientation, and support for innovation (Beaulieu et al., 2014; Ouwens et al., 2008). This instrument has been validated in many populations, countries, and organizational contexts including hospital and community-based health and social services, and primary care. Face and content validity were rigorously established at the time of development. TCI is among the few instruments that have been validated and used in a variety of contexts and countries (Lemieux-Charles & McGuire, 2006). TCI has been validated in different languages, and the four-factor structure has always been confirmed (Strating & Nieboer, 2009). Higher performance on the TCI has been associated with improved health outcomes, better access to care, improved patient satisfaction, improved job satisfaction, and openness to innovation (Lemieux-Charles & McGuire, 2006; Tseng et al., 2009).

#### Team Emergency Assessment Measure (TEAM)

The TEAM instrument uses a 5-point scale and covers three categories: leadership, teamwork and task management (Cooper, Cant et al., 2010). Encompassed within these categories are nine elements—leadership control, communication, team climate, adaptability, situation awareness (perception), situation awareness (projection); prioritization, clinical standards, as well as co-operation and co-ordination. TEAM was found to be a valid and reliable instrument and should be a useful addition to clinicians’ instrument set for the measurement of teamwork during medical emergencies. The content, construct and concurrent validity, internal consistency, inter-rater reliability, re-test reliability and feasibility ratings all had satisfactory levels. Although the instrument was primarily designed for cardiac resuscitation teams, it has also been found to be a valid measure for teams managing simulated patients who are deteriorating and is likely to be of use to trauma and medical emergency teams (Cooper et al., 2013).

## Discussion

The goal of this research was to conduct review of psychometric evidence to identify the most robust instruments and provide an overview of the properties and limitations of these instruments. As health care professionals continue to work collaboratively, it is important to effectively evaluate health care teams in an effort to identify successful models of care and improve existing models. Hundreds of surveys have been developed to measure different types of health care teams; however, this has led to an overwhelming amount of surveys, the majority of which have not been validated.

Although several surveys were identified in this research, seven are arguably the most frequently identified in the literature, the practicality of these surveys remains in question. Beyond simply measuring the number of times these surveys were used as a measure of psychometric evidence, the viability and practicality of these tools is also examined. For example, CPAT has 56 questions. With a time-constraint workload for the health care professionals, filling the survey can be time consuming and the quality of the responses may also be affected by the high number of questions. Reducing the number of questions without losing the validity of the surveys would provide an efficient manner in which health care professionals can fill out the survey. Similarly, the original ICU N-P-Q is a 120-item scale with an 81-item revised and shortened version. Some researchers suggest that training the person who is applying the survey is required due to the complexity of using the instrument to assess the team (Undre et al., 2007). This makes the instrument impractical and limits the use for health care teams or researchers.

Different dimensions of teamwork were considered across surveys, which also provides insight to the underlying assumptions of the theoretical underpinnings of the instruments. Understanding the dimensions of teamwork and the theoretical underpinnings of the instrument are very important given their influences on what measures are used in understanding teamwork performance (Anderson & West, 1998). For example, those that want to understand teamwork performance as modeled by the partnership synergy framework should not use TCI or Edmondson’s psychological safety questionnaire because these two instruments base their teamwork performance on psychological safety and group climate for innovation (Anderson & West, 1998; Edmondson, 1999). Likewise, those who believe psychological safety is a key component of teamwork should not use CPAT because it does not measure any form of psychological safety (Schroder et al., 2011).

Based on our review, we suggest that CPAT provides the best option when the goal is to measure teamwork in a general health care setting. The dimensions are derived from current literature and it is one of few surveys that includes patient involvement as one of the dimensions of teamwork. Although there are 56 questions plus three additional open-ended questions, it provides the most comprehensive evaluation of a health care team. For those specifically seeking to assess health care teams in operating rooms, the OTAS is recommended (Undre et al., 2007). The use of the OTAS tool, however, has practical challenges given the training requirements prior to use. The TCI instrument is recommended if psychological safety in teamwork is a high priority. TCI has been validated numerous times and has multiple versions in different languages. There are also different versions with varying lengths. TCI is highly respected and recommended when measuring teamwork in general health care settings (Beaulieu et al., 2014).

Although some surveys include a patient dimension within the teamwork domains, the patient dimension is still missing in most instruments. This is a large gap in team evaluation. Recent literature suggests that patients are essential and valid members of the health care team and should be included in all aspects of patient care (LaDonna et al., 2017). The CPAT instrument includes patients as one of the dimensions to assess teamwork, however, the target recipients of the surveys are health care professionals and not patients (Schroder et al., 2011). Given the importance of patient-centered care for health care delivery and the shift towards an egalitarian relationship between patients and health care providers (Fix et al., 2018), we contend that patient experience should be included as an important dimension that contributes to teamwork assessment. Adapting instruments to include the patient is essential due to the importance of patients, their families and their caregivers as important contributors to health care teams (McMillan et al., 2013).

Although the literature suggests that teams do not necessarily have to be co-located to be successful, the majority of surveys in this review assume that the teams are co-located and bounded (i.e., consistent membership). More specifically, surveys are often limited to only core clinical teams or contingency teams formed during emergencies, and rarely ever include other non-clinical members as part of the team. As a result, surveys can be limited in function and may not capture the performance of teamwork in larger unbounded teams or teams across different departments or sectors. The usefulness and practicalities of instruments need to be considered given current system transformation towards integrated care with large, cross-sector collaboration.

### Limitations

In this study, efforts to reduce bias were made throughout our study by having multiple researchers assess the inclusion of potential articles. Two independent researchers used the ROBIS checklist to establish inter-rater reliability.

Given that we extracted data from systematic reviews, information in scoping reviews, as well as surveys created in the recent years, may not have been identified during the data extraction process. Although it is possible that this study has not identified every existing survey in the literature, we are confident that robust instruments reported in systematic reviews have been identified, which was the primary goal of this study.

Another limitation was our approach to counting and reporting instruments. Counting the frequency in which the instrument is mentioned in the systematic reviews may not suggest that the instrument is the best or optimal. It is possible that newly created surveys are better with stronger validations. We assumed that instruments frequently identified were more robust. Our threshold of four references to be included in the final reporting may omit valid instruments. As an example, the Assessment of Interprofessional Team Collaboration Scale (AITCS; Orchard et al., 2012) has 37 items and measures partnership, cooperation, and coordination. It has good psychometric properties and includes questions on patient involvement. A revised 23-item version of AITCS is also valid and reliable (Orchard et al., 2018); AITCS has been translated into an Italian version with promising signs of validity (Caruso et al., 2018). However, newly developed instruments like the AITCS would not have had enough time for exposure to be identified in a systematic review. Even if a systematic review had identified them, the limited time period would have limited the number of references.

### Future Research

Future research should aim to include a higher number of systematic reviews that capture the instruments that provide the highest evidence on measuring teamwork. This is extremely important because existing surveys are often revised through further application and also translated to different languages, which can further validate the survey. Despite this study only observing systematic reviews, there were well over 100 surveys identified. Researchers suggest that existing surveys should be revised and tested in different health care settings. In practice, many ignore this and create new surveys. This raises another challenge making current literature even more difficult to navigate with so many instruments existing. Future research should aim to take existing instruments and modify them to meet the context of specific teams and settings. In addition, improving the availability and open access of instruments should be considered.

## Conclusion

In this study, we conducted a review of psychometric evidence to identify robust instruments in the literature that measure teamwork in health care settings and report on their theoretical underpinnings, psychometric properties, limitations, and practicality. Rather than offering a long list of instruments relevant in a particular health care setting, our paper only included instruments that have been considered robust across several systematic reviews and relevant to measure teamwork in a variety of health care settings.

This review of psychometric evidence was focused on the syntheses provided in published systematic reviews as systematic reviews and meta-analyses are considered the highest level of available evidence. Identifying robust instruments that measure teamwork can potentially be useful for researchers and clinicians who seek to assess teamwork in a variety of clinical settings and health care teams. Selecting which instrument to use will be dependent on context as well as preference toward theoretical underpinnings. More research is needed to both understand how to incorporate the patient dimension, as well as how to adapt instruments for use in larger unbounded teams.

## Supplemental Material

Supplemental Material, sj-docx-1-ehp-10.1177_01632787211040859 - Interdisciplinary Health Care Evaluation Instruments: A Review of Psychometric Evidence Click here for additional data file. Supplemental Material, sj-docx-1-ehp-10.1177_01632787211040859 for Interdisciplinary Health Care Evaluation Instruments: A Review of Psychometric Evidence by Hosung (Joel) Kang, Cecilia Flores-Sandoval, Benson Law and Shannon Sibbald in Evaluation & the Health Professions

Supplemental Material, sj-docx-2-ehp-10.1177_01632787211040859 - Interdisciplinary Health Care Evaluation Instruments: A Review of Psychometric Evidence Click here for additional data file. Supplemental Material, sj-docx-2-ehp-10.1177_01632787211040859 for Interdisciplinary Health Care Evaluation Instruments: A Review of Psychometric Evidence by Hosung (Joel) Kang, Cecilia Flores-Sandoval, Benson Law and Shannon Sibbald in Evaluation & the Health Professions

## References

[bibr1-01632787211040859] Aksnes D. W. Langfeldt L. Wouters P. (2019). Citations, citation indicators, and research quality: An overview of basic concepts and theories. SAGE Open, 9(1), 215824401982957. 10.1177/2158244019829575

[bibr2-01632787211040859] Anderson N. R. West M. A . (1998). Measuring climate for work group innovation: Development and validation of the team climate inventory. Journal of Organizational Behavior, 19(3), 235–258. 10.1002/(SICI)1099-1379(199805)19:3<235::AID-JOB837>3.0.CO;2-C

[bibr3-01632787211040859] Beaulieu M.-D. Dragieva N. Del Grande C. Dawson J. Haggerty J. L. Barnsley J. Hogg W. E Tousignant P West M. A . (2014). The team climate inventory as a measure of primary care teams’ processes: Validation of the French version. Healthcare Policy = Politiques De Sante, 9(3), 40–54. 24726073 PMC3999572

[bibr4-01632787211040859] Bookey-Bassett S. Markle-Reid M. Mckey C. A. Akhtar-Danesh N. (2016). Understanding interprofessional collaboration in the context of chronic disease management for older adults living in communities: A concept analysis. Journal of Advanced Nursing, 73(1), 71–84. 10.1111/jan.13162 27681818

[bibr5-01632787211040859] Boult C. Boult L. B. Morishita L. Dowd B. Kane R. L. Urdangarin C. F. (2001). A randomized clinical trial of outpatient geriatric evaluation and management. Journal of the American Geriatrics Society, 49(4), 351–359. 10.1046/j.1532-5415.2001.49076.x 11347776

[bibr6-01632787211040859] Brinkman W. B. Geraghty S. R. Lanphear B. P. Khoury J. C. Gonzalez del Rey J. A. DeWitt T. G. Britto M. T. (2006). Evaluation of resident communication skills and professionalism: A matter of perspective? Pediatrics, 118(4), 1371–1379. 10.1542/peds.2005-3214 17015525

[bibr7-01632787211040859] Buist M. D. Moore G. E. Bernard S. A. Waxman B. P. Anderson J. N. Nguyen T. B . (2002). Effects of a medical emergency team on reduction of incidence of and mortality from unexpected cardiac arrests in hospital: Preliminary study. BMJ, 324(7334), 387–390. 10.1136/bmj.324.7334.387 11850367 PMC65530

[bibr8-01632787211040859] Caruso R. Magon A. Dellafiore F. Griffini S. Milani L. Stievano A. Orchard C. A. (2018). Italian version of the assessment of Interprofessional Team Collaboration Scale II (I-AITCS II): A multiphase study of validity and reliability amongst healthcare providers. La Medicina Del Lavoro, 109(4), 316–324. 10.23749/mdl.v109i4.7101 30168504 PMC7682163

[bibr9-01632787211040859] Clary-Muronda V. Pope C. (2016). Integrative review of instruments to measure team performance during neonatal resuscitation simulations in the birthing room. Journal of Obstetric, Gynecologic & Neonatal Nursing, 45(5), 684–698. 10.1016/j.jogn.2016.04.007 27470178

[bibr10-01632787211040859] Cooper S. Cant R. Porter J. Sellick K. Somers G. Kinsman L. Nestel D. (2010). Rating medical emergency teamwork performance: Development of the team emergency assessment measure (TEAM). Resuscitation, 81(4), 446–452. 10.1016/j.resuscitation.2009.11.027 20117874

[bibr11-01632787211040859] Cooper S. Endacott R. Cant R. (2010). Measuring non-technical skills in medical emergency care: A review of assessment measures. Open Access Emergency Medicine: OAEM, 2, 7–16. 27147832 10.2147/oaem.s6693 PMC4806821

[bibr12-01632787211040859] Cooper S. Porter J. Peach L. (2013). Measuring situation awareness in emergency settings: A systematic review of tools and outcomes. Open Access Emergency Medicine: OAEM, 6, 1–7. 10.2147/OAEM.S53679 27147872 PMC4753990

[bibr13-01632787211040859] Dougherty M. B. Larson E. (2005). A review of instruments measuring nurse-physician collaboration. The Journal of Nursing Administration, 35(5), 244–253. 15891488 10.1097/00005110-200505000-00008

[bibr14-01632787211040859] Durieux N. Vandenput S. Pasleau F. (2013). OCEBM levels of evidence system. Revue Medicale De Liege, 68(12), 644–649. 24564030

[bibr15-01632787211040859] Edmondson A. (1999). Psychological safety and learning behavior in work teams. Administrative Science Quarterly, 44(2), 350–383. 10.2307/2666999

[bibr16-01632787211040859] Fix G. M. VanDeusen Lukas C. Bolton R. E. Hill J. N. Mueller N. LaVela S. L. Bokhour B. G. (2018). Patient-centred care is a way of doing things: How healthcare employees conceptualize patient-centred care. Health Expectations, 21(1), 300–307. 10.1111/hex.12615 28841264 PMC5750758

[bibr17-01632787211040859] Fransen A. F. de Boer L. Kienhorst D. Truijens S. E. van Runnard Heimel P. J. Oei S. G. (2017). Assessing teamwork performance in obstetrics: A systematic search and review of validated tools. European Journal of Obstetrics, Gynecology, and Reproductive Biology, 216, 184–191. 10.1016/j.ejogrb.2017.06.034 28787688

[bibr18-01632787211040859] Gellis Z. D. Kim E. Hadley D. Packel L. Poon C. Forciea M. A Bradway C Streim J Seman J Hayden T Johnson J . (2019). Evaluation of interprofessional health care team communication simulation in geriatric palliative care. Gerontology & Geriatrics Education, 40(1), 30–42. 10.1080/02701960.2018.1505617 30160623

[bibr19-01632787211040859] Gosselin É. Marceau M. Vincelette C. Daneau C.-O. Lavoie S. Ledoux I. (2019). French translation and validation of the Mayo high performance teamwork scale for nursing students in a high-fidelity simulation context. Clinical Simulation in Nursing, 30, 25–33. 10.1016/j.ecns.2019.03.002

[bibr20-01632787211040859] Havyer R. D. A. Nelson D. R. Wingo M. T. Comfere N. I. Halvorsen A. J. McDonald F. S. Reed D. A. (2016). Addressing the interprofessional collaboration competencies of the association of American medical colleges: A systematic review of assessment instruments in undergraduate medical education. Academic Medicine, 91(6): 865–888. 26703415 10.1097/ACM.0000000000001053

[bibr21-01632787211040859] Havyer R. D. A. Wingo M. T. Comfere N. I. Nelson D. R. Halvorsen A. J. McDonald F. S. Reed D. A. (2014). Teamwork assessment in internal medicine: A systematic review of validity evidence and outcomes. Journal of General Internal Medicine, 29(6), 894–910. 10.1007/s11606-013-2686-8 24327309 PMC4026505

[bibr22-01632787211040859] Hutchison B. Glazier R. (2013). Ontario’s primary care reforms have transformed the local care landscape, but a plan is needed for ongoing improvement. Health Affairs, 32(4), 695–703. 10.1377/hlthaff.2012.1087 23569049

[bibr23-01632787211040859] Jacob J. Boshoff K. Stanley R. Stewart H. Wiles L. (2017). Interprofessional collaboration within teams comprised of health and other professionals: A systematic review of measurement tools and their psychometric properties. Internet Journal of Allied Health Sciences and Practice, 15(2), 8.

[bibr80-01632787211040859] Kobayashi R. McAllister C. A. (2014). Similarities and differences in perspectives on interdisciplinary collaboration among hospice team members. American Journal of Hospice and Palliative Medicine®, 31(8), 825–832. 10.1177/1049909113503706 24113193

[bibr24-01632787211040859] LaDonna K. A. Bates J. Tait G. R. McDougall A. Schulz V. Lingard L ., & Heart Failure/Palliative Care Teamwork Research Group. (2017). “Who is on your health-care team?” Asking individuals with heart failure about care team membership and roles. Health Expectations: An International Journal of Public Participation in Health Care and Health Policy, 20(2), 198–210. 10.1111/hex.12447 26929430 PMC5354030

[bibr25-01632787211040859] Langhorne P. Duncan P. (2001). Does the organization of postacute stroke care really matter? Stroke, 32(1), 268–274. 11136947 10.1161/01.str.32.1.268

[bibr26-01632787211040859] Lasker R. D. Weiss E. S. Miller R . (2001). Partnership synergy: A practical framework for studying and strengthening the collaborative advantage. The Milbank Quarterly, 79(2), 179–205, III–IV. 11439464 10.1111/1468-0009.00203 PMC2751192

[bibr27-01632787211040859] Lemieux-Charles L. McGuire W. L. (2006). What do we know about health care team effectiveness? A review of the literature. Medical Care Research and Review: MCRR, 63(3), 263–300. 10.1177/1077558706287003 16651394

[bibr28-01632787211040859] Litwin M. S. Fink A. (2003). How to assess and interpret survey psychometrics (Vol. 8). Sage.

[bibr29-01632787211040859] Malec J. F. Torsher L. C. Dunn W. F. Wiegmann D. A. Arnold J. J. Brown D. A. Phatak V. (2007). The Mayo High Performance Teamwork Scale: Reliability and validity for evaluating key crew resource management skills. Simulation in Healthcare: The Journal of the Society for Simulation in Healthcare, 2(1), 4–10. 10.1097/SIH.0b013e31802b68ee 19088602

[bibr30-01632787211040859] McMillan S. S. Kendall E. Sav A. King M. A. Whitty J. A. Kelly F. Wheeler A. J. (2013). Patient-centered approaches to health care: A systematic review of randomized controlled trials. Medical Care Research and Review, 70(6), 567–596. 10.1177/1077558713496318 23894060

[bibr31-01632787211040859] Moher D. Liberati A. Tetzlaff J. Altman D. G. Group T. P. (2009). Preferred reporting items for systematic reviews and meta-analyses: The PRISMA statement. PLOS Medicine, 6(7), e1000097. 10.1371/journal.pmed.1000097 19621072 PMC2707599

[bibr32-01632787211040859] Mokkink L. B. Terwee C. B. Patrick D. L. Alonso J. Stratford P. W. Knol D. L. Bouter L. M de Vet H. C. W . (2010). The COSMIN checklist for assessing the methodological quality of studies on measurement properties of health status measurement instruments: An international Delphi study. Quality of Life Research: An International Journal of Quality of Life Aspects of Treatment, Care and Rehabilitation, 19(4), 539–549. 10.1007/s11136-010-9606-8 PMC2852520 20169472

[bibr33-01632787211040859] Morey J. C. Simon R. Jay G. D. Wears R. L. Salisbury M. Dukes K. A. Berns S. D. (2002). Error reduction and performance improvement in the emergency department through formal teamwork training: Evaluation results of the MedTeams project. Health Services Research, 37(6), 1553–1581. 12546286 10.1111/1475-6773.01104 PMC1464040

[bibr34-01632787211040859] O’Leary K. J. Ritter C. D. Wheeler H. Szekendi M. K. Brinton T. S. Williams M. V. (2010). Teamwork on inpatient medical units: Assessing attitudes and barriers. Quality & Safety in Health Care, 19(2), 117–121. 10.1136/qshc.2008.028795 20351159

[bibr35-01632787211040859] Oliver D. P. Wittenberg-Lyles E. M. Day M. (2007). Measuring interdisciplinary perceptions of collaboration on hospice teams. American Journal of Hospice and Palliative Medicine®, 24(1), 49–53. 10.1177/1049909106295283 17347505

[bibr36-01632787211040859] Onwochei D. N. Halpern S. Balki M. (2017). Teamwork assessment tools in obstetric emergencies: A systematic review. Simulation in Healthcare: Journal of the Society for Simulation in Healthcare, 12(3), 165–176. 10.1097/SIH.0000000000000210 28009653

[bibr37-01632787211040859] Orchard C. A. King G. A. Khalili H. Bezzina M. B. (2012). Assessment of Interprofessional Team Collaboration Scale (AITCS): Development and testing of the instrument. Journal of Continuing Education in the Health Professions, 32(1), 58–67. 10.1002/chp.21123 22447712

[bibr38-01632787211040859] Orchard C. A. Pederson L. L. Read E. Mahler C. Laschinger H. (2018). Assessment of Interprofessional Team Collaboration Scale (AITCS): Further testing and instrument revision. Journal of Continuing Education in the Health Professions, 38(1), 11–18. 10.1097/CEH.0000000000000193 29517613

[bibr39-01632787211040859] Ouwens M. Hulscher M. Akkermans R. Hermens R. Grol R. Wollersheim H. (2008). The Team Climate Inventory: Application in hospital teams and methodological considerations. Quality and Safety in Health Care, 17(4), 275–280. 10.1136/qshc.2006.021543 18678725

[bibr40-01632787211040859] Palumbo R. (2017). Examining the impacts of health literacy on healthcare costs: An evidence synthesis. Health Services Management Research, 30(4), 197–212. 10.1177/0951484817733366 29034727

[bibr41-01632787211040859] Paterson M. L. Medves J. Dalgarno N. O’Riordan A. Grigg R. (2013). The timely open communication for patient safety project. Journal of Research in Interprofessional Practice and Education, 3(1), 22–24.

[bibr42-01632787211040859] Rosenman E. D. Ilgen J. S. Shandro J. R. Harper A. L. Fernandez R. (2015). A systematic review of tools used to assess team leadership in health care action teams. Academic Medicine, 90(10), 1408–1422. 10.1097/ACM.0000000000000848 26200585

[bibr43-01632787211040859] Rosser W. W. Colwill J. M. Kasperski J. Wilson L. (2011). Progress of Ontario’s family health team model: A patient-centered medical home. The Annals of Family Medicine, 9(2), 165–171. 10.1370/afm.1228 21403144 PMC3056865

[bibr44-01632787211040859] Rydenfält C. Odenrick P. Larsson P. A. (2017). Organizing for teamwork in healthcare: An alternative to team training? Journal of Health Organization and Management, 31(3), 347–362. 10.1108/JHOM-12-2016-0233 28686132

[bibr45-01632787211040859] Schroder C. Medves J. Paterson M. Byrnes V. Chapman C. O’Riordan A Pichora D Kelly C . (2011). Development and pilot testing of the collaborative practice assessment tool. Journal of Interprofessional Care, 25(3), 189–195. 10.3109/13561820.2010.532620 21182434

[bibr46-01632787211040859] Shoemaker S. J. Parchman M. L. Fuda K. K. Schaefer J. Levin J. Hunt M. Ricciardi R. (2016). A review of instruments to measure interprofessional team-based primary care. Journal of Interprofessional Care, 30(4), 423–432. 10.3109/13561820.2016.1154023 27212003

[bibr47-01632787211040859] Shortell S. M. Rousseau D. M. Gillies R. R. Devers K. J. Simons T. L. (1991). Organizational assessment in intensive care units (ICUs): Construct development, reliability, and validity of the ICU nurse-physician questionnaire. Medical Care, 29(8), 709–726. 1875739 10.1097/00005650-199108000-00004

[bibr48-01632787211040859] Strating M. M. Nieboer A. P. (2009). Psychometric test of the Team Climate Inventory-Short Version investigated in Dutch quality improvement teams. BMC Health Services Research, 9(1), 126. 10.1186/1472-6963-9-126 19627621 PMC2724501

[bibr49-01632787211040859] Tseng H.-M. Liu F.-C. West M. A. (2009). The Team Climate Inventory (TCI): A Psychometric test on a Taiwanese sample of work groups. Small Group Research, 40(4), 465–482. 10.1177/1046496409334145

[bibr50-01632787211040859] Undre S. Sevdalis N. Healey A. N. Darzi A. Vincent C. A. (2007). Observational teamwork assessment for surgery (OTAS): Refinement and application in urological surgery. World Journal of Surgery, 31(7), 1373–1381. 10.1007/s00268-007-9053-z 17487527

[bibr51-01632787211040859] Valentine M. A. Nembhard I. M. Edmondson A. C. (2015). Measuring teamwork in health care settings: A review of survey instruments. Medical Care, 53(4), e16–30. 10.1097/MLR.0b013e31827feef6 24189550

[bibr52-01632787211040859] Walters S. J. Stern C. Robertson-Malt S. (2016). The measurement of collaboration within healthcare settings: A systematic review of measurement properties of instruments. JBI Database of Systematic Reviews and Implementation Reports, 14(4), 138–197. 10.11124/JBISRIR-2016-2159 27532315

[bibr53-01632787211040859] Whiting P. Savović J. Higgins J. P. Caldwell D. M. Reeves B. C. Shea B. Davies P. Kleijnen J. Churchill R ., & ROBIS Group. (2016). ROBIS: A new tool to assess risk of bias in systematic reviews was developed. Journal of Clinical Epidemiology, 69, 225–234. 10.1016/j.jclinepi.2015.06.005 26092286 PMC4687950

[bibr54-01632787211040859] Whittaker G. Abboudi H. Khan M. S. Dasgupta P. Ahmed K. (2015). Teamwork assessment tools in modern surgical practice: A systematic review. Surgery Research and Practice, 2015, 494827. 10.1155/2015/494827 26425732 PMC4573989

