# Supplementary material for: Interdisciplinary Health Care Evaluation Instruments: A Review of Psychometric Evidence
Source: Eval Health Prof. 2021 Aug 19;45(3):223–34. doi: 10.1177/01632787211040859 (PMC9446429; doi:10.1177/01632787211040859)
Supplement: Supplemental Material, sj-docx-1-ehp-10.1177_01632787211040859 - Interdisciplinary Health Care Evaluation Instruments: A Review of Psychometric Evidence [file sj-docx-1-ehp-10.1177_01632787211040859.docx]

**Appendix A**

**List of included reviews**

1. Bookey-Bassett S, Markle-Reid M, McKey C, Akhtar-Danesh N. A review of instruments to measure interprofessional collaboration for chronic disease management for community-living older adults. *J Interprof Care*. 2016;30(2):201-210.

2. Clary-Muronda V, Pope C. Integrative review of instruments to measure team performance during neonatal resuscitation simulations in the birthing room. *JOGNN - J Obstet Gynecol Neonatal Nurs*. 2016;45:684-698.

3. Cooper S, Endacott R, Cant R. Measuring non-technical skills in medical emergency care: A review of assessment measures. *Open Access Emerg Med*. 2010;2:7-16. http://www.dovepress.com/getfile.php?fileID=5701.

4. Cooper S, Porter J, Peach L. Measuring situation awareness in emergency settings: A systematic review of tools and outcomes. *Open Access Emerg Med*. 2013;6:1-7. doi:10.2147/OAEM.S53679

5. Dougherty MB, Larson E. A review of instruments measuring nurse-physician collaboration. *J Nurs Adm*. 2005;35(5):244-253. doi:10.1097/00005110-200505000-00008

6. Fransen AF, de Boer L, Kienhorst D, Truijens SE, van Runnard Heimel PJ, Oei SG. Assessing teamwork performance in obstetrics. *Eur J Obstet Gynecol Reprod Biol*. 2017;216:184-191.

7. Havyer RD, Wingo MT, Comfere NI, et al. Teamwork assessment in internal medicine: A systematic review of validity evidence and outcomes. *J Gen Intern Med*. 2014;29(6):894-910.

8. Havyer, RD., Nelson, DR., Wingo, MT., Comfere, NI., Halvorsen, AJ., McDonald, FS., & Reed, DA. Addressing the Interprofessional Collaboration Competencies of the Association of American Medical Colleges: a systematic review of assessment instruments in undergraduate medical education. *Acad Med.* 2016; 91(6): 865-888.

9. Jacob J, Boshoff K, Stanley R, Stewart H, Wiles L. Interprofessional collaboration within teams comprised of health and other professionals: a systematic review of measurement tools and their psychometric properties. *Internet J Allied Heal Sci Pract*. 2017;15(2).

10. Onwochei DN, Halpern S, Balki M. Teamwork assessment tools in obstetric emergencies: A systematic review. *Simul Healthc*. 2017;12(3):165-176. doi:10.1097/SIH.0000000000000210

11. Rosenman ED, Ilgen JS, Shandro JR, et al. A systematic review of tools used to assess team leadership in health care action teams. *Acad Med*. 2015;90(10):1408-1422. doi:http://dx.doi.org/10.1097/ACM.0000000000000848

12. Shoemaker SJ, Parchman ML, Kerwin Fuda K, et al. A review of instruments to measure interprofessional team-based primary care. *J Interprof Care*. 2016;30(4):423-432.

13. Valentine MA, Nembhard IM, Edmondson AC. Measuring teamwork in health care settings: a review of survey instruments. *Med Care*. 2015;53(4):e16-e30. doi:10.1097/MLR.0b013e31827feef6

14. Walters SJ, Stern, C, Robertson-Malt S. The measurement of collaboration within healthcare settings; a systematic review of measurement properties of instruments. *JBI Database Syst Rev Implement Reports*. 2016;14(4):138-197. [doi:10.11124/JBISRIR-2016-2159](https://doi.org/10.11124/JBISRIR-2016-2159)

15. Whittaker G, Abboudi H, Shamim Khan M, Dasgupta P, Ahmed K. Teamwork assessment tools in modern surgical practice: A systematic review. *Surg Res Pract*. 2015:494827.
