# Supplementary material for: Interdisciplinary Health Care Evaluation Instruments: A Review of Psychometric Evidence
Source: Eval Health Prof. 2021 Aug 19;45(3):223–34. doi: 10.1177/01632787211040859 (PMC9446429; doi:10.1177/01632787211040859)
Supplement: Supplemental Material, sj-docx-2-ehp-10.1177_01632787211040859 - Interdisciplinary Health Care Evaluation Instruments: A Review of Psychometric Evidence [file sj-docx-2-ehp-10.1177_01632787211040859.docx]

|  | Appendix B – Data Extraction Table of Instruments | | | | | | | | | | | | | | | | | | |
| --- | --- | --- | --- | --- | --- | --- | --- | --- | --- | --- | --- | --- | --- | --- | --- | --- | --- | --- | --- |
| Author | | Instrument Name | Bookey-Bassett et al. (2016) | Clary-Muronda & Pope (2016) | Cooper et al. (2010) | Cooper et al. (2013) | Fransen et al. (2017) | Havyer et al. (2014) | Havyer et al. (2016) | Rosenman et al. (2015) | Valentine et al. (2015) | Whittaker et al. (2015) | Onwochi et al. (2017) | Jacob et al. (2017) | Walters et al. (2016) | Dougherty et al, (2005) | Shoemaker et al. (2016) | Count |  |
| Vanhaecht et al. (2007) | | Care Process Self-Evaluation Tool (CPSET) |  |  |  |  |  |  |  |  |  |  |  |  | X |  |  | 1 |  |
| Ohman-Strickland et al. (2007) | | “Using Learning Teams for Reflective Adaptation” or ULTRA Survey |  |  |  |  |  |  |  |  |  |  |  |  |  |  | X | 1 |  |
| Temkin-Greener et al. (2004) | | Adapted ICU Nurse Physician Questionnaire |  |  |  |  |  |  |  |  |  |  |  | X |  |  | X | 2 |  |
| Kolb (1995) | | Adapted Leadership Behavior Description Questionnaire (LBDQ) |  |  | X |  |  |  |  | X |  |  |  |  |  |  |  | 2 |  |
| Fletcher et al. (2003); Jankouskas et al. (2011) | | Anaesthetists' Nontechnical Skills (ANTS) |  |  |  | X |  | X | X |  |  |  |  |  |  |  |  | 3 |  |
| Curran et al. (2011); Heinemann et al. (1999) | | Attitudes Toward Health Care Teams |  |  |  |  |  | X | X |  |  |  |  |  |  |  | X | 3 |  |
| Wright et al. (2009) | | Behaviorally-Anchored Team Skill Rating Scale |  |  |  |  |  |  | X |  |  |  |  |  |  |  |  | 1 |  |
| Guise et al. (2008) | | Clinical Teamwork Skills (CTS) |  |  |  |  | X |  |  | X |  |  | X |  |  |  |  | 3 |  |
| Baggs (1994) | | Collaboration and Satisfaction about Care Decisions |  |  |  |  |  |  |  |  | X |  |  |  |  | X |  | 2 |  |
| Kahn and McDough (1997) | | Collaboration Scale |  |  |  |  |  |  |  |  | X |  |  |  |  |  |  | 1 |  |
| Masse et al. (2008) | | Collaboration Scale |  |  |  |  |  |  |  |  | X |  |  |  |  |  |  | 1 |  |
| Hollar et al. (2012) | | Collaborative Healthcare Interdisciplinary Relationship Planning (CHIRP) Scale |  |  |  |  |  |  | X |  |  |  |  |  |  |  |  | 1 |  |
| Schroder et al. (2011) | | Collaborative Practice Assessment Tool (CPAT) | X |  |  |  |  |  |  |  | X |  |  | X | X |  |  | 4 |  |
| Weiss and Davis (1985) | | Collaborative Practice Scale (CPS) |  |  |  |  |  |  |  |  |  |  |  |  |  | X |  | 1 |  |
| Frankel et al. (2007) | | Communication and Teamwork Skills (CATS) Assessment |  |  |  |  |  |  | X |  |  |  |  |  |  |  | X | 2 |  |
| Monge et al. (1981) | | Communication Competency Questionnaire |  |  | X |  |  |  |  |  |  |  |  |  |  |  |  | 1 |  |
| Loughry et al. (2007) | | Comprehensive Assessment of Team Member Effectiveness |  |  |  |  |  |  |  |  |  |  |  |  |  |  | X | 1 |  |
| Alexander et al. (2005) | | Cross Functional Team Processes |  |  |  |  |  |  |  |  | X |  |  |  |  |  |  | 1 |  |
| Pinto et al. (1993) | | Cross-Functional Cooperation |  |  |  |  |  |  |  |  | X |  |  |  |  |  |  | 1 |  |
| Berendsen et al. (2010) | | Doctor’s Opinion on Collaboration (DOC) |  |  |  |  |  |  |  |  |  |  |  |  | X |  |  | 1 |  |
| Youngblood et al. (2008) | | Emergency Medicine Crisis Resource Management Scale (EMRCM) |  |  |  |  |  |  | X | X |  |  |  |  |  |  |  | 2 |  |
| Bradley et al. (2009); Cooper et al. (2007) | | Emergency Team Dynamics (ETD) Scale |  |  | X |  |  |  | X |  |  |  |  |  |  |  |  | 2 |  |
| Sudikoff et al. (2009) | | Global Competency Score (GCS) |  |  |  |  |  |  |  | X |  |  |  |  |  |  |  | 1 |  |
| Kim et al. (2009); Morgan et al. (2007) | | Global Rating Scale (GRS) |  |  |  |  | X |  |  | X |  |  | X |  |  |  |  | 3 |  |
| Vinokur-Kaplan (1995) | | Group Effectiveness Interdisciplinary Collaboration |  |  |  |  |  |  |  |  | X |  |  |  |  |  |  | 1 |  |
| Peterson (2012) | | Group Emotional Intelligence Individual Regulation (GEIQ–IR) Scale |  |  |  |  |  |  |  |  |  |  |  |  |  |  | X | 1 |  |
| Slack et al. (2001) | | Group Growth Evaluation Form |  |  |  |  |  |  | X |  |  |  |  |  |  |  |  | 1 |  |
| Sorra and Nieva (2004) | | Hospital Survey on Patient Safety |  |  |  |  |  |  |  |  | X |  |  |  |  |  |  | 1 |  |
| Morgan et al. (2007) | | Human Factors Rating Scale (HFRS) |  |  |  |  | X |  |  | X |  |  | X |  |  |  |  | 3 |  |
| Mellin et al. (2003) | | Index of Interprofessional Team Collaboration for Expanded School Mental Health (IITC-ESMH) |  |  |  |  |  |  |  |  |  |  |  | X | X |  |  | 2 |  |
| Nuno-Solinis et al. (2013) | | Inter-Professional Collaboration Between Two Different Levels of Care (IPC-DLC) |  |  |  |  |  |  |  |  |  |  |  |  | X |  |  | 1 |  |
| Basran et al. (2012) | | Interdisciplinary Education Perception Scale (IEPS) |  |  |  |  |  |  | X |  |  |  |  |  |  |  |  | 1 |  |
| Dadiz et al. (2013) | | Interdisciplinary Simulation-Based Training to Improve Birthing Room Communication |  | X |  |  |  |  |  |  |  |  |  |  |  |  |  | 1 |  |
| Cameron et al. (2009) | | Interprofessional Attitudes Questionnaire (IAQ) |  |  |  |  |  |  | X |  | X |  |  |  |  |  |  | 2 |  |
| Curran et al. (2011) | | Interprofessional Collaborator Assessment Rubric (ICAR) |  |  |  |  |  |  | X |  |  |  |  |  |  |  |  | 1 |  |
| King et al. (2010) | | Interprofessional Socialization and Valuing Scale (ISVS) |  |  |  |  |  |  | X |  |  |  |  |  | X |  |  | 2 |  |
| Korner and Wirtz (2013) | | Internal Participation Scale (IPS) |  |  |  |  |  |  |  |  |  |  |  |  | X |  |  | 1 |  |
| Hojat et al. (1999, 2012); Ward et al. (2008) | | Jefferson Scale of Attitudes Toward Physician–Nurse Collaboration |  |  |  |  |  | X | X |  |  |  |  |  |  | X |  | 3 |  |
| Jones and Barry (2011) | | Jones Synergy Scale |  |  |  |  |  |  |  |  |  |  |  | X |  |  |  | 1 |  |
| Jones and Barry (2011) | | Jones Trust Scale |  |  |  |  |  |  |  |  |  |  |  | X |  |  |  | 1 |  |
| Sigalet et al. (2013) | | KidSIM Team Performance Scale Checklist |  | X |  |  |  |  |  |  |  |  |  |  |  |  |  | 1 |  |
| Grant et al. (2012) | | Leadership and Communication Skills (LCS) |  |  |  |  |  |  |  |  |  |  |  |  |  |  | X | 1 |  |
| Carlson et al. (2009) | | Leadership and Team Behavior Management Tool |  |  |  |  |  |  | X |  |  |  |  |  |  |  |  | 1 |  |
| van Beuzekom et al. (2007) | | Leiden Opening Theater and Intensive Care Safety (LOTICS) |  |  |  |  |  |  |  |  | X |  |  |  |  |  | X | 2 |  |
| Malec et al. (2007); Garbee et al. (2013) | | Mayo High Performance Teamwork Scale (MHPTS) |  |  | X |  |  |  | X | X |  |  |  |  |  |  | X | 4 |  |
| Hall et al. (2011) | | McMaster– Ottawa Team Observed Structured Clinical Encounter (TOSCE) Observer Score Sheet |  |  |  |  |  |  | X |  |  |  |  |  |  |  |  | 1 |  |
| Weaver et al. (2010) | | Medical Performance Assessment Tool for Communication and Teamwork (MedPACT) |  |  |  |  |  |  |  | X |  |  |  |  |  |  |  | 1 |  |
| Rodgers et al. (2010) | | Advanced Cardiovascular Life Support Mega Code Performance Score Sheet (ACLS) |  |  |  |  |  |  |  | X |  |  |  |  |  |  |  | 1 |  |
| Parker Oliver et al. (2007); Wittenberg-Lyles et al. (2010) | | Modified Index of Interdisciplinary Collaboration (MIIC) | X |  |  |  |  |  | X |  |  |  |  | X | X |  |  | 4 |  |
| Mazur et al. (1979) | | Modified Team Opinion Questionnaire |  |  |  |  |  |  | X |  |  |  |  |  |  |  |  | 1 |  |
| Calhoun et al. (2011) | | Multi-rater Team Performance During Simulated Crisis Instrument (TPDSCI) |  | X |  |  |  |  |  |  |  |  |  |  |  |  |  | 1 |  |
| Kenaszchuk, Reeves et al. (2010) | | Multiple Group Measurement Scale (MGMS) |  |  |  |  |  |  |  |  |  |  |  |  | X |  |  | 1 |  |
| Violato et al. (2003) | | Multisource Feedback (MSF) |  |  |  |  |  |  |  |  |  | X |  |  |  |  |  | 1 |  |
| Jukkala and Henly (2007) | | Neonatal Resuscitation Experience Index |  | X |  |  |  |  |  |  |  |  |  |  |  |  |  | 1 |  |
| Amin et al. (2013) | | Neonatal Resuscitation Simulation Self-Assessment Questionnaire |  | X |  |  |  |  |  |  |  |  |  |  |  |  |  | 1 |  |
| Meier et al. (2012) | | Non-technical Skills Scale (NOTECHS) |  |  |  |  |  |  | X |  |  |  |  |  |  |  |  | 1 |  |
| Yule et al. (2006; 2008) | | Nontechnical Skills for Surgeons (NOTSS) |  |  |  | X |  |  |  | X |  | X |  |  |  |  |  | 3 |  |
| Steinmann et al. (2011) | | Nontechnical Skills System Modified for trauma (T NOTECH) |  |  |  |  |  |  |  | X |  |  |  |  |  |  |  | 1 |  |
| Ushiro (2009) | | Nurse-Physician Collaboration Scale |  |  |  |  |  |  |  |  | X |  |  |  |  |  | X | 2 |  |
| Shortell et al. (1991) | | ICU Nurse-Physician Questionnaire |  |  |  |  |  | X |  |  | X |  |  | X |  | X |  | 4 |  |
| Adams et al. (1995) | | Nurses Opinion Questionnaire (NOQ) |  |  |  |  |  |  |  |  |  |  |  |  |  | X |  | 1 |  |
| Kalisch et al. (2010); Kalisch and Lee (2013) | | Nursing Teamwork Survey |  |  |  |  |  |  |  |  | X |  |  |  |  |  | X | 2 |  |
| Walker et al. (2011) | | Observational Skill-Based Clinical Assessment Instrument for Resuscitation (OSCAR) |  |  |  |  |  |  |  | X |  |  |  |  |  |  |  | 1 |  |
| Healey at al. (2004); Undre et al. (2007) | | Observational Teamwork Assessment for Surgery (OTAS) |  |  |  |  |  |  |  | X |  | X | X |  | X |  |  | 4 |  |
| Tregunno et al. (2009); Morgan et al. (2012) | | Obstetric Team Performance (AOTP) |  |  |  |  | X |  |  | X |  |  | X |  |  |  |  | 3 |  |
| Paige et al. (2014) | | Operating Room Teamwork Assessment Scale (ORTAS) |  |  |  |  |  |  | X |  |  |  |  |  |  |  |  | 1 |  |
| Wallin et al. (2007) | | Operating Team Resource Management Survey (OTRMS) |  |  |  |  |  |  | X |  |  |  |  |  |  |  |  | 1 |  |
| Passauler-Baierl et al. (2014) | | Observational Teamwork Assessment for Surgery Tool (OTAS-D) |  |  |  |  |  |  |  |  |  |  |  |  | X |  |  | 1 |  |
| Brannik et al. (1993) | | Overall Performance on Simulator Task |  |  |  |  |  |  |  |  | X |  |  |  |  |  |  | 1 |  |
| Mishra et al. (2009) | | Oxford Nontechnical Skills System (NOTECHS) |  |  |  | X |  |  |  |  |  | X |  |  |  |  | X | 3 |  |
| Weiss et al. (2002) | | Partnership Self-Assessment Tool (PSAT) | X |  |  |  |  |  |  |  |  |  |  |  |  |  |  | 1 |  |
| Henry et al. (2013) | | Patients’ Insights and Views Observing Teams (PIVOT)Ssurvey |  |  |  |  |  |  |  |  |  |  |  |  |  |  | X | 1 |  |
| Rousseau et al. (2012) | | Perception of Interprofessional Collaboration Model PINCOM-Q REVISED |  |  |  |  |  |  |  |  |  |  |  | X |  |  |  | 1 |  |
| Odegard (2006) | | Perception of Interprofessional Collaboration Model Questionnaire  (PINCOM-Q) |  |  |  |  |  |  |  |  |  |  |  | X |  |  |  | 1 |  |
| Copnell et al. (2004) | | Perceptions About Interdisciplinary Collaboration Scale |  |  |  |  |  |  |  |  | X |  |  |  |  |  | X | 2 |  |
| Curran et al. (2012) | | Perceptions of Effective Interprofessional Teams Scale |  |  |  |  |  |  | X |  |  |  |  |  |  |  |  | 1 |  |
| Lockyer et al. (2006) | | Performance Checklist to Assess Neonatal Resuscitation Mega Code Skill |  | X |  |  |  |  |  |  |  |  |  |  |  |  |  | 1 |  |
| Nagpal et al. (2011) | | Postoperative Handover Assessment Tool (PoHAT) |  |  |  |  |  |  |  | X |  |  |  |  |  |  |  | 1 |  |
| de Wet et al. (2010) | | Primary Care Patient Safety Climate |  |  |  |  |  |  |  |  | X |  |  |  |  |  | X | 2 |  |
| Erickson et al. (2004) | | Professional Practice Environment Revised Scale (PPE) |  |  |  |  |  |  |  |  |  |  |  | X |  |  |  | 1 |  |
| Adams et al. (1995) | | Professional Working Relationships |  |  |  |  |  |  |  |  | X |  |  |  |  |  |  | 1 |  |
| Edmonson (1999) | | Psychological Safety and Teamwork Learning |  |  |  |  |  |  |  |  | X |  |  |  |  |  | X | 2 |  |
| Wauben et al. (2011) | | Questionnaire Perception of Communication, Teamwork and Situation Awareness |  |  |  |  |  |  |  |  |  |  |  |  |  |  | X | 1 |  |
| Atack et al. (2009) | | Readiness for Interprofessional Learning Scale (RIPLS) |  |  |  |  |  |  | X |  |  |  |  |  |  |  |  | 1 |  |
| Gittell (2002) | | Relational Coordination |  |  |  |  |  |  |  |  | X |  |  |  |  |  |  | 1 |  |
| Sexton et al. (2006) | | Safety Attitudes Questionnaire |  |  |  |  |  | X |  |  | X |  |  |  |  |  | X | 3 |  |
| Hojat et al. (20120 | | Scale of Attitudes Towards Physician–Pharmacist Collaboration |  |  |  |  |  |  | X |  |  |  |  |  |  |  |  | 1 |  |
| van der Heide et al. (2006) | | Scoring Instrument for the Assessment of Neonatal Resuscitation Skills |  | X |  |  |  |  |  |  |  |  |  |  |  |  |  | 1 |  |
| Mitchell et al. (2013) | | Scrub Practitioners List of Intraoperative Nontechnical Skills (SPLINTS) |  |  |  |  |  |  |  |  |  | X |  |  |  |  |  | 1 |  |
| Morgan et al. (2015) | | Situation Awareness Global Assessment Scale (SAGAT) |  |  |  | X |  |  |  |  |  |  | X |  |  |  |  | 2 |  |
| Hänsel et al. (2012) | | Situation Awareness Global Assessment Technique (SAGAT) |  |  |  |  |  |  | X |  |  |  |  |  |  |  |  | 1 |  |
| Hobgood et al. (2010) | | Standardized Patient Evaluation (SPE) of Teamwork Skills Performance |  |  |  |  |  |  | X |  |  |  |  |  |  |  |  | 1 |  |
| Posmontier et al. (2012) | | Team Attitudes Questionnaire (TAQ) |  |  |  |  |  |  | X |  |  |  | X |  |  |  |  | 2 |  |
| Anderson and West (1998) | | Team Climate Inventory (TCI) | X |  | X |  |  | X |  |  | X |  |  |  |  |  | X | 5 |  |
| Batorowicz and Sheperd (2008) | | Team Decision Making Questionnaire (TDMQ) |  |  |  |  |  |  |  |  |  |  |  | X |  |  | X | 2 |  |
| Wageman et al. (2005) | | Team Diagnostic Survey |  |  |  |  |  |  |  |  | X |  |  |  |  |  | X | 2 |  |
| Curran et al. (2005) | | Team Dynamics Observation Checklist |  |  |  |  |  |  | X |  |  |  |  |  |  |  |  | 1 |  |
| Pearce and Sims (2002) | | Team Effectiveness |  |  |  |  |  |  |  |  | X |  |  |  |  |  | X | 2 |  |
| Cooper et al. (2010) | | Team Emergency Assessment Measure (TEAM) |  |  |  | X |  |  |  | X | X |  | X |  |  |  |  | 4 |  |
| Strasser et al. (2002) | | Team Functioning |  |  |  |  |  |  |  |  | X |  |  |  |  |  |  | 1 |  |
| Robertson et al. (2010) | | Team Knowledge Test |  |  |  |  |  |  | X |  |  |  |  |  |  |  |  | 1 |  |
| Seers (1989) | | Team Member Exchange (TMX) |  |  |  |  |  |  |  |  | X |  |  |  |  |  |  | 1 |  |
| Fernandez Castelao et al. (2011) | | Team Member Verbalization |  |  |  |  |  |  | X |  |  |  |  |  |  |  |  | 1 |  |
| La Duckers et al. (2008) | | Team Organization |  |  |  |  |  |  |  |  | X |  |  |  |  |  |  | 1 |  |
| Slack et al. (2001) | | Team Orientation and Behavior Inventory |  |  |  |  |  |  | X |  |  |  |  |  |  |  |  | 1 |  |
| Thompson et al. (2009) | | Team Performance Scale (TPS) |  |  |  |  |  |  | X |  |  |  |  |  |  |  |  | 1 |  |
| Doolen et al. (2003) | | Team Process |  |  |  |  |  |  |  |  | X |  |  |  |  |  | X | 2 |  |
| Denison et al. (1996) | | Team Process Domain |  |  |  |  |  |  |  |  | X |  |  |  |  |  | X | 2 |  |
| Hauptman and Hirji (1999) | | Team Process Quality |  |  |  |  |  |  |  |  | X |  |  |  |  |  |  | 1 |  |
| Robertson et al. (2010) | | Team Skills Checklist Video Rating |  |  |  |  |  |  | X |  |  |  |  |  |  |  |  | 1 |  |
| Curran et al. (2005) | | Team Skills Scale |  |  |  |  |  |  | X |  |  |  |  |  |  |  |  | 1 |  |
| Millward and Jeffries (2001) | | Team Survey |  |  |  |  |  |  |  |  | X |  |  |  |  |  | X | 2 |  |
| Senior and Swailes (2007) | | Teamwork Survey |  |  |  |  |  |  |  |  | X |  |  |  |  |  | X | 2 |  |
| Meier et al. (2012) | | TeamSTEPPS Knowledge Exam |  |  |  |  |  |  | X |  |  |  |  |  |  |  |  | 1 |  |
| Agency for Healthcare Research and Quality (2014) | | TeamSTEPPS Teamwork Perceptions Questionnaire (T-TPQ) |  | X |  |  |  |  |  |  |  |  |  |  |  |  |  | 1 |  |
| Garbee et al. (2013) | | Teamwork Assessment Scale (TAS) |  |  |  |  |  |  | X |  |  |  |  |  |  |  |  | 1 |  |
| Frengley et al. (2011) | | Teamwork Behavioral Rate (TBR) |  |  |  |  |  |  |  | X |  |  |  |  |  |  |  | 1 |  |
| Chesluk et al. (2012) | | Teamwork Effectiveness Assessment Module (TEAM) |  |  |  |  |  |  |  |  |  |  |  |  |  |  | X | 1 |  |
| Mayer et al. (2011) | | Teamwork Evaluation of Nontechnical Skills (TENTS) |  |  |  |  |  |  |  | X |  |  |  |  |  |  |  | 1 |  |
| Qvist et al. (2010) | | Teamwork Failure Prevention Questionnaire (TFP) Questionnaire |  |  |  |  |  |  |  |  |  |  |  |  |  |  | X | 1 |  |
| MacDonnell et al. (2012) | | Teamwork Global Rating Scale |  |  |  |  |  |  | X |  |  |  |  |  |  |  |  | 1 |  |
| Wholey et al. (2012) | | Teamwork in Assertive Community Treatment Scale (TACT) |  |  |  |  |  |  |  |  |  |  |  | X |  |  |  | 1 |  |
| Siassakos et al. (2011) | | Teamwork Measurement Tool |  |  |  |  | X |  |  |  |  |  | X |  |  |  |  | 2 |  |
| Hoegl and Gemeunden (2001) | | Teamwork Quality Survey |  |  |  |  |  |  |  |  | X |  |  |  |  |  | X | 2 |  |
| Friesen et al. (2008) | | Teamwork Scale |  |  |  |  |  |  |  |  | X |  |  |  |  |  | X | 2 |  |
| Hutchinson et al. (2006) | | Teamwork Scale |  |  |  |  |  |  |  |  | X |  |  |  |  |  |  | 1 |  |
| Ottestad et al. (2007) | | Technical and Non-Technical Rating Scale for Septic Shock |  |  | X |  |  |  |  |  |  |  |  |  |  |  |  | 1 |  |
| Orchard et al. (2012) | | The Assessment of Interprofessional Team Collaboration Scale (AITCS) | X |  |  |  |  |  |  |  |  |  |  |  | X |  | X | 3 |  |
| Upenieks et al. (2010) | | The Healthcare Team Vitality Instrument (HTVI) |  |  |  |  |  |  |  |  |  |  |  |  | X |  | X | 2 |  |
| Kim et al. (2006) | | The Ottawa Crisis Resource Management Global Rating Scale |  |  | X |  |  |  |  |  |  |  |  |  |  |  |  | 1 |  |
| Holcomb et al. (2002) | | The Trauma Team Evaluation Tool |  |  | X |  |  |  |  |  |  |  |  |  |  |  |  | 1 |  |
| Finley et al. (2013) | | The Work Relationships Scale (WRS) |  |  |  |  |  |  |  |  |  |  |  |  |  |  | X | 1 |  |
| Catchpole et al. (2007) | | Tool for Resuscitation Assessment Using Computerized Simulation (TRACS) |  |  |  |  |  |  |  | X |  |  |  |  |  |  |  | 1 |  |
| Capella et al. (2010) | | Trauma Team Performance (TPOT) |  |  |  |  |  |  |  | X |  |  |  |  |  |  |  | 1 |  |
| Warrier et al. (2013) | | Value of Teams Survey |  |  |  |  |  |  | X |  |  |  |  |  |  |  |  | 1 |  |
| Curran et al. (2005) | | Weekly Team Inventory |  |  |  |  |  |  | X |  |  |  |  |  |  |  |  | 1 |  |
| Campion et al. (1993) | | Work Group Effectiveness |  |  |  |  |  |  |  |  | X |  |  |  |  |  | X | 2 |  |
